# Cholesterol metabolism shapes immune low-response states in LUAD: a multi-omics cholesterol metabolism signature predicts immunotherapy benefit and identifies DHCR7 as a therapeutic target

**DOI:** 10.3389/fimmu.2025.1696360

**Published:** 2025-10-30

**Authors:** Mingjun Du, Jianan Zheng, Guangyao Zhou, Yu Zhuang, Chenjun Huang, Wei Ye

**Affiliations:** ^1^ Department of Thoracic Surgery, The First Affiliated Hospital with Nanjing Medical University, Nanjing, China; ^2^ Department of Lung Cancer, Tianjin Lung Cancer Center, National Clinical Research Center for Cancer, Key Laboratory of Cancer Prevention and Therapy, Tianjin’s Clinical Research Center for Cancer, Tianjin Medical University Cancer Institute and Hospital, Tianjin, China; ^3^ Department of Thoracic Surgery, Nanjing Chest Hospital, Nanjing, China; ^4^ Afliated Nanjing Brain Hospital, Nanjing Medical University, Nanjing, China

**Keywords:** lung adenocarcinoma, cholesterol metabolism, DHCR7, immune microenvironment, immunotherapy

## Abstract

**Background:**

Cholesterol metabolism has been shown to affect the tumor microenvironment in various cancers, but its immunological role in lung adenocarcinoma (LUAD) remains unclear.

**Methods:**

We integrated 1,682 LUAD samples (including 7 treatment-naïve bulk cohorts and 3 immunotherapy bulk cohorts) to develop a Cholesterol Metabolism Signature (CMS) based on cholesterol metabolism-associated genes. Survival analysis, ROC curves, and PCA were used to evaluate the ability of CMS to predict prognosis and immunotherapy efficacy. Immune infiltration analysis, single-cell transcriptomics, as well as *in vitro* and *in vivo* experiments were further performed to investigate the function and mechanism of the key CMS gene, DHCR7.

**Results:**

CMS effectively predicted the survival outcomes and immunotherapy benefits of LUAD patients, which was consistently validated in all independent cohorts. Patients with high CMS had worse prognosis. Compared with 51 previously published LUAD signatures, CMS showed higher predictive accuracy and stratification ability. Immune-related analyses showed that the high CMS group had reduced immune cell infiltration and suppressed immune function, which was further supported by single-cell analysis revealing enhanced immunosuppressive pathways. Expression of the key gene DHCR7 was highly correlated with CMS score (R = 0.42, P<0.05), negatively associated with many immune-related genes and immune cycles, and promoted poor prognosis and cancer pathways. Multiplex immunohistochemistry confirmed that regions with high DHCR7 expression had fewer infiltrating CD8T and CD20B cells. *In vitro* experiments demonstrated that silencing DHCR7 inhibited the proliferation, invasion, and migration of LUAD cells; mouse models confirmed that suppressing DHCR7 enhanced the efficacy of PD-1 inhibitors. Flow cytometry showed that DHCR7 knockdown significantly increased IFN-γ+CD8T and GZMB+CD8T cell infiltration.

**Conclusion:**

Our study demonstrates that the CMS can effectively predict prognosis and immunotherapy response in LUAD. DHCR7, as a key gene in CMS, is closely related to immune suppression and poor prognosis. Inhibition of DHCR7 can improve the tumor immune microenvironment and enhance the efficacy of immunotherapy, suggesting that DHCR7 is a potential new target for LUAD immunotherapy.

## Introduction

Lung cancer remains the leading cause of cancer incidence and mortality worldwide, posing a serious threat to human health (1). Lung adenocarcinoma (LUAD) is the most common histological subtype of lung cancer, and its incidence continues to rise (2, 3). Although traditional treatments such as surgery, chemotherapy, and radiotherapy have progressed in recent years, the overall prognosis for advanced LUAD remains unsatisfactory. Immunotherapy, particularly immune checkpoint inhibitors (ICIs) targeting programmed cell death protein 1 (PD-1) or its ligand PD-L1, has revolutionized the treatment of non-small cell lung cancer (NSCLC), especially LUAD, significantly prolonging survival in a subset of patients (4–6). As immunotherapy becomes a standard treatment option, the therapeutic paradigm for patients with LUAD is undergoing profound change. However, the clinical benefit of ICIs still varies greatly among individuals; some patients exhibit limited or no response to immunotherapy, and adverse events remain a concern (7). Numerous studies have shown that immunosuppression, impaired immune cell infiltration, and immune tolerance within the tumor microenvironment are key factors affecting immunotherapy efficacy (8, 9). Therefore, exploring the molecular mechanisms regulating the tumor immune microenvironment and identifying biomarkers that can accurately predict immunotherapy outcomes and guide patient stratification are important focal points in both clinical and basic LUAD research.

In recent years, cancer cell metabolic reprogramming has been recognized as a crucial mechanism regulating the tumor immune microenvironment, with different metabolic pathways exerting profound effects on immune responses (10, 11). In particular, aberrant cholesterol metabolism not only provides the substrate for membrane synthesis and key signaling molecules in tumor cells but also shapes an immunosuppressive microenvironment by modulating the metabolism and function of immune cells such as T cells, B cells, and macrophages, thereby influencing immunotherapy efficacy (12–14). Recent studies have also found that the UPR key factor XBP1 in cancer cells can promote cholesterol synthesis and package it into small extracellular vesicles, which are taken up by MDSCs via macropinocytosis, thereby shaping an immunosuppressive microenvironment and weakening the efficacy of immunotherapy (15). Although some scholars have investigated the role of cholesterol metabolism in tumorigenesis and progression (16, 17), systematic studies on its function in the immune microenvironment and immunotherapy of LUAD—as well as molecular markers and key regulatory genes involved-remain limited.

With the advancement of high-throughput sequencing and bioinformatics analysis, constructing molecular scoring systems based on specific biological pathways, combined with clinical data and multi-omics analysis, not only improves the accuracy of patient prognosis prediction but also provides new strategies for optimizing immunotherapy screening and stratification (18–21). Studies have indicated that DHCR7, a critical gene in cholesterol biosynthesis, is closely related to immune regulation in certain malignancies (22, 23), but its specific role and regulatory mechanisms in LUAD remain to be elucidated.

In this study, we focused on LUAD, integrating large-scale, multicenter datasets to systematically construct and validate a Cholesterol Metabolism Signature (CMS). We comprehensively evaluated the value of CMS in prognosis and immunotherapy outcome prediction, and further focused on DHCR7 to explore its pivotal role in regulating the tumor immune microenvironment and influencing immunotherapy response in LUAD. Our aim is to provide a theoretical and practical basis for precise stratification management and novel therapeutic targets in LUAD immunotherapy.

## Method

### Datasets and cholesterol metabolism-related gene sets

The gene expression data, somatic single nucleotide variant (SNV) data, and copy number variation (CNV) data used in this study were all obtained from The Cancer Genome Atlas (TCGA) database. Normal lung tissue expression data were obtained from the Genotype-Tissue Expression (GTEx) database for subsequent differential expression analysis. In addition, we integrated six independent LUAD cohorts from the Gene Expression Omnibus (GEO) database, including GSE13213 (24) (n=117), GSE26939 (25) (n=115), GSE29016 (26) (n=39), GSE30219 (27) (n=85), GSE31210 (28) (n=226), and GSE42127 (29) (n=133). Batch effects among different cohorts were corrected using the ComBat algorithm, followed by normalization to ensure consistency across datasets. To evaluate the clinical utility of cholesterol metabolism-related gene sets in immunotherapy, we further included eight NSCLC immunotherapy cohorts: POPLAR (30) (n=59), OAK (30) (n=257), NG (31) (n=46), GSE126044 (32) (n=16), GSE135222 (33) (n=27), GSE166449 (34) (n=22), and GSE207422 (35) (n=24). The cholesterol metabolism-related gene set was obtained from the GeneCards database (https://www.genecards.org/) by searching with the keyword “cholesterol metabolism” and selecting genes with a Relevance Score ≥15. The final set of core genes related to cholesterol metabolism was used for subsequent analyses.

### Differential expression and prognostic modeling

To examine expression differences, the limma package was utilized to identify cholesterol metabolism-related genes with significant dysregulation between tumor and control tissues, using thresholds of FDR < 0.05 and |log2FC| > 1. For mutation landscape characterization, the maftools package (36) was employed to comprehensively visualize the distribution of mutations across these genes, with Oncoplot used to display representative genes harboring high mutation frequencies and to summarize dominant mutation patterns. For prognostic biomarker development, univariate Cox regression was first applied to highlight genes closely correlated with survival outcomes. A range of statistical and machine learning algorithms were then tested through ten-fold cross-validation, including Lasso, Ridge, CoxBoost, Random Survival Forest (RSF), Enet, GBM, stepwise Cox regression, SuperPC, plsRcox, and survival-SVM, with performance mainly evaluated using the concordance index (C-index). The final prognostic signature’s predictive power was assessed via Kaplan-Meier survival analysis, principal component analysis (PCA), and time-dependent ROC curves, and benchmarked against published models.

### Immune microenvironment profiling

Immune phenotyping of samples was conducted using immunophenoscore (IPS) data from The Cancer Immunome Atlas platform (37), providing a quantitative estimate of the likelihood of immunotherapy responsiveness. The activity of immune cells and related pathways in the tumor microenvironment was assessed using the ssGSEA approach. Additionally, comprehensive immune infiltration data for TCGA samples were compiled through the TIMER2.0 platform (38), integrating results from various computational methods for a systematic view of the immune landscape. The immune-related genes are summarized from previous literature (39).

### Single-cell transcriptome analysis

Single-cell gene expression analysis was based on raw data obtained from the Genome Sequence Archive for Human (GSA for Human) of the China National Center for Bioinformation (CNCB, https://ngdc.cncb.ac.cn/gsa-human/, accession number HRA005794). Seurat was used for initial quality control and processing (40). Inclusion required each gene to be detected in at least 10 cells within a given cluster. Cells with fewer than 200 or more than 5,000 detected genes, or with mitochondrial UMI fractions exceeding 10%, were excluded. Dataset integration across samples was accomplished with the harmony algorithm. Subsequent analysis involved identifying highly variable genes, conducting principal component analysis (PCA), and applying t-SNE on the top 30 principal components for dimensionality reduction. Marker genes for each subset were determined using the FindAllMarkers function, and cell types were annotated based on established lineage markers from previous literature.

### Cell-cell communication network

In this study, the CellChat R package (41) was employed for an in-depth analysis of single-cell RNA sequencing data, aiming to elucidate the signaling networks between different cell types within the tumor microenvironment. Quality control, normalization, clustering, and cell type annotation were first performed on the single-cell expression data. Subsequently, based on the built-in ligand-receptor database in CellChat, cell–cell communication pathways among different cell subpopulations were inferred. Combined with the expression profiles of signature genes in each population, major signaling sources, receptors, and corresponding signaling axes were identified. Network visualization techniques were used to present the communication strength, key signaling pathways, and critical nodes between cell populations, thereby enabling further analysis of their biological significance and roles during tumor progression.

### Sample collection

All subjects in this study were LUAD patients treated at the Department of Pathology, Tianjin Medical University Cancer Institute and Hospital. All samples were derived from formalin-fixed, paraffin-embedded (FFPE) surgical specimens. None of the patients had received radiotherapy, chemotherapy, immunotherapy, or targeted therapy before surgery. The pathological diagnosis of each specimen was confirmed by at least two experienced pathologists. All procedures strictly adhered to medical ethical standards and were approved by the Institutional Ethics Committee (Approval No.: bc2023152). Written informed consent was obtained from all participants.

### Multiplex immunofluorescence staining and analysis

FFPE tissue sections (approximately 4 μm in thickness) were subjected to standard clinical protocols for deparaffinization: sequential incubation in xylene and graded ethanol solutions, followed by rehydration in distilled water. After high-temperature antigen retrieval with citrate buffer and cooling to room temperature, the sections were incubated in 5% goat serum for 30 minutes at room temperature to block nonspecific binding sites. Subsequently, primary antibodies were applied sequentially and incubated overnight at 4 °C in a humidified chamber: DHCR7 (ab226784, 1:500), CD4 (ab133616, 1:500), CD8 (ab217344, 1:2000), and CD20 (ab64088, 1:100), all prepared according to the respective datasheets. After incubation, sections were rinsed multiple times in PBS buffer. Fluorophore-conjugated secondary antibodies corresponding to each primary antibody were then added and incubated at room temperature for 1 hour in the dark. After further PBS washing, cell nuclei were counterstained with DAPI at room temperature for 10 minutes, followed by a brief rinse with distilled water. Finally, slides were air-dried and mounted with an antifade mounting medium. Imaging was performed using a multichannel laser confocal fluorescence microscope, with high-resolution images acquired for each fluorophore channel. All procedures for antibody incubation, washing, and imaging were carried out strictly in accordance with standardized experimental protocols.

### Cell culture and RNA interference

A549 and PC9 cell lines were obtained from a certified cell bank and maintained in RPMI 1640 medium supplemented with 10% fetal bovine serum and antibiotics at 37 °C in a humidified incubator with 5% CO_2_. To modulate DHCR7 gene expression, specific shRNA sequences were delivered to target cells using a lentiviral vector system. Following puromycin selection, quantitative real-time PCR was performed to validate knockdown efficiency.

### Cell proliferation assay

Cellular proliferation was evaluated using the CCK-8 assay. Both cell lines were digested with trypsin, counted, and then seeded into 96-well plates according to experimental grouping, comprising knockdown, vector control, and blank control groups, with multiple replicates for each. After cells adhered for 24 hours, CCK-8 solution was added at various time points. Absorbance was measured using a microplate reader, and proliferation curves were plotted for group comparison.

### Establishment and intervention of subcutaneous tumor model in mice

For *in vivo* experiments, healthy female C57BL/6 mice were randomly assigned to groups. A total of 1×10^6^ LLC NC or sh−Dhcr7–treated cells suspended in sterile PBS were injected subcutaneously at a single site on the right dorsal flank (one site per mouse). Tumor length and width were measured daily with calipers to monitor growth. When any tumor reached a volume of 100 mm³ (designated as day 0), mice received intraperitoneal injections of either a PD−1 monoclonal antibody or an IgG2a isotype control (100 μg per mouse) on days 1, 4, and 7. On day 12 after the first intervention, euthanasia was performed using cervical dislocation, carried out rapidly (approximately 1–2 seconds) by trained personnel. Death was confirmed by cessation of respiration and heartbeat and loss of the righting reflex, after which tumors were harvested, their volumes and weights measured, and samples processed for immunological analyses.

### Flow cytometry analysis

For flow cytometry, freshly excised tumors were minced and digested in a solution containing collagenase IV and DNase I to prepare single-cell suspensions. The cell suspensions were filtered to remove debris and immune cells were enriched with density gradient centrifugation. Sorted cells were incubated with stimulants to induce functional molecule expression, followed by Fc receptor blocking to minimize non-specific binding. Surface staining was performed with fluorescence-conjugated antibodies against CD45, CD3e, and CD8α. After fixation and permeabilization, intracellular staining was conducted using antibodies against IFNγ and GZMB. Data were acquired using a flow cytometer, and sequential gating was used to identify CD45^+^ leukocytes, CD3^+^ T cells, CD8^+^ T cells, and to assess the proportion of IFNγ and GZMB positive cells within the CD8^+^ T cell population.

### Statistical analysis

All data analyses were performed using R software. Comparisons between two groups were conducted using t-tests or non-parametric tests, and comparisons among multiple groups were conducted using analysis of variance. Kaplan-Meier method and Log-rank test were used for survival curve comparison. Correlation analyses were performed using Spearman correlation coefficients. All statistical tests were two-sided, and p < 0.05 was considered statistically significant.

## Result

### Mutation characteristics and prognostic implications of cholesterol-related genes

**Figure 1:**
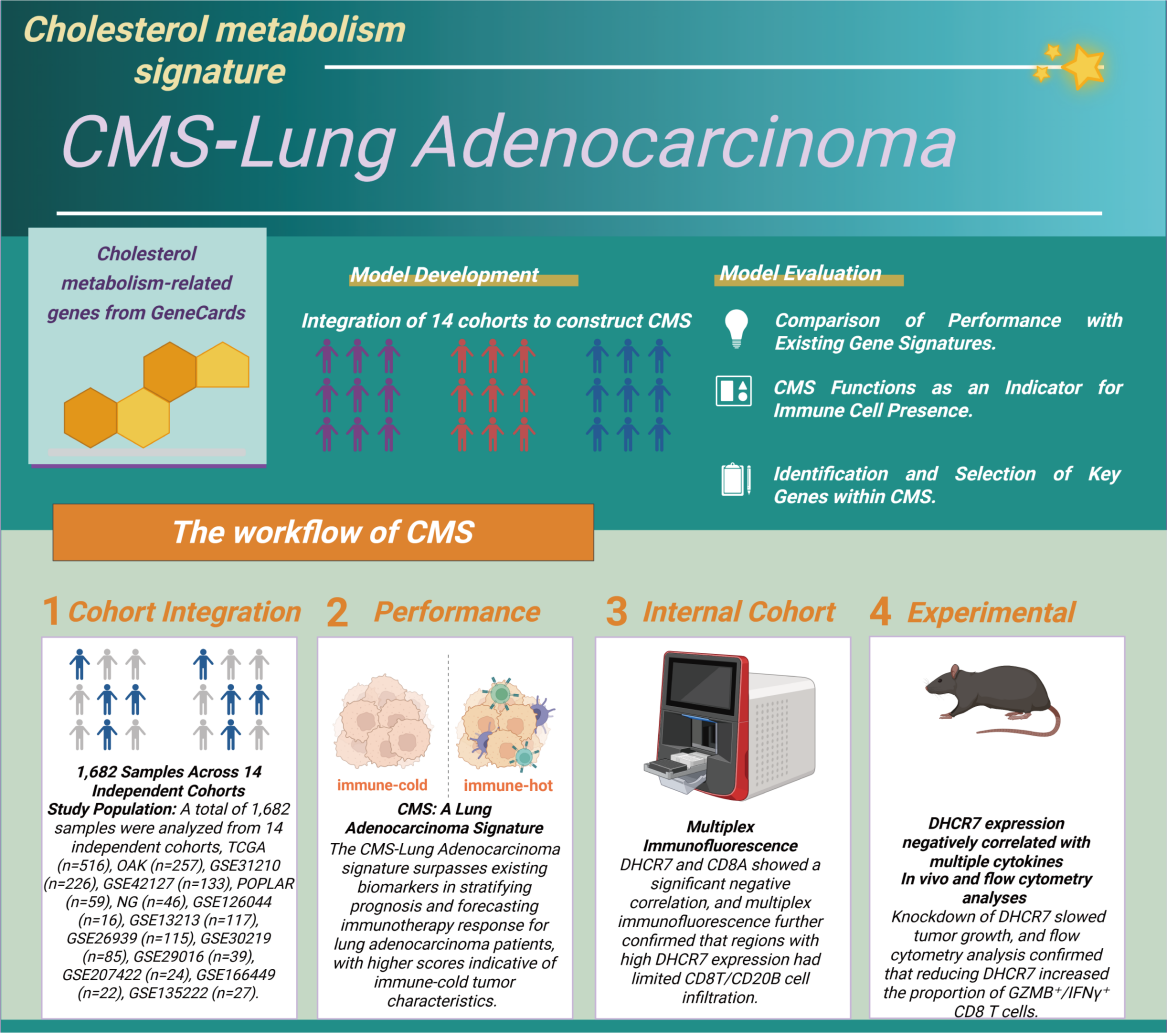
Construction and validation process of the cholesterol metabolism signature (CMS). We integrated data from 1,682 lung adenocarcinoma samples across 14 independent cohorts to identify cholesterol metabolism-related genes and developed the CMS scoring system. In the evaluation phase, we validated the CMS in three main areas (1): comparing its predictive ability for prognosis and immunotherapy with other gene models; (2) assessing its effectiveness as a marker for immune cell infiltration; (3) identifying key genes. The specific steps include: (1) integrating sample data; (2) evaluating the CMS performance in predicting prognosis and immunotherapy response; (3) experimentally verifying the relationship between DHCR7 and immune cell infiltration; (4) confirming that knocking down DHCR7 increases the infiltration of GZMB+ CD8+ T cells and enhances the effectiveness of PD-1 blockade.

**Figure 2:**
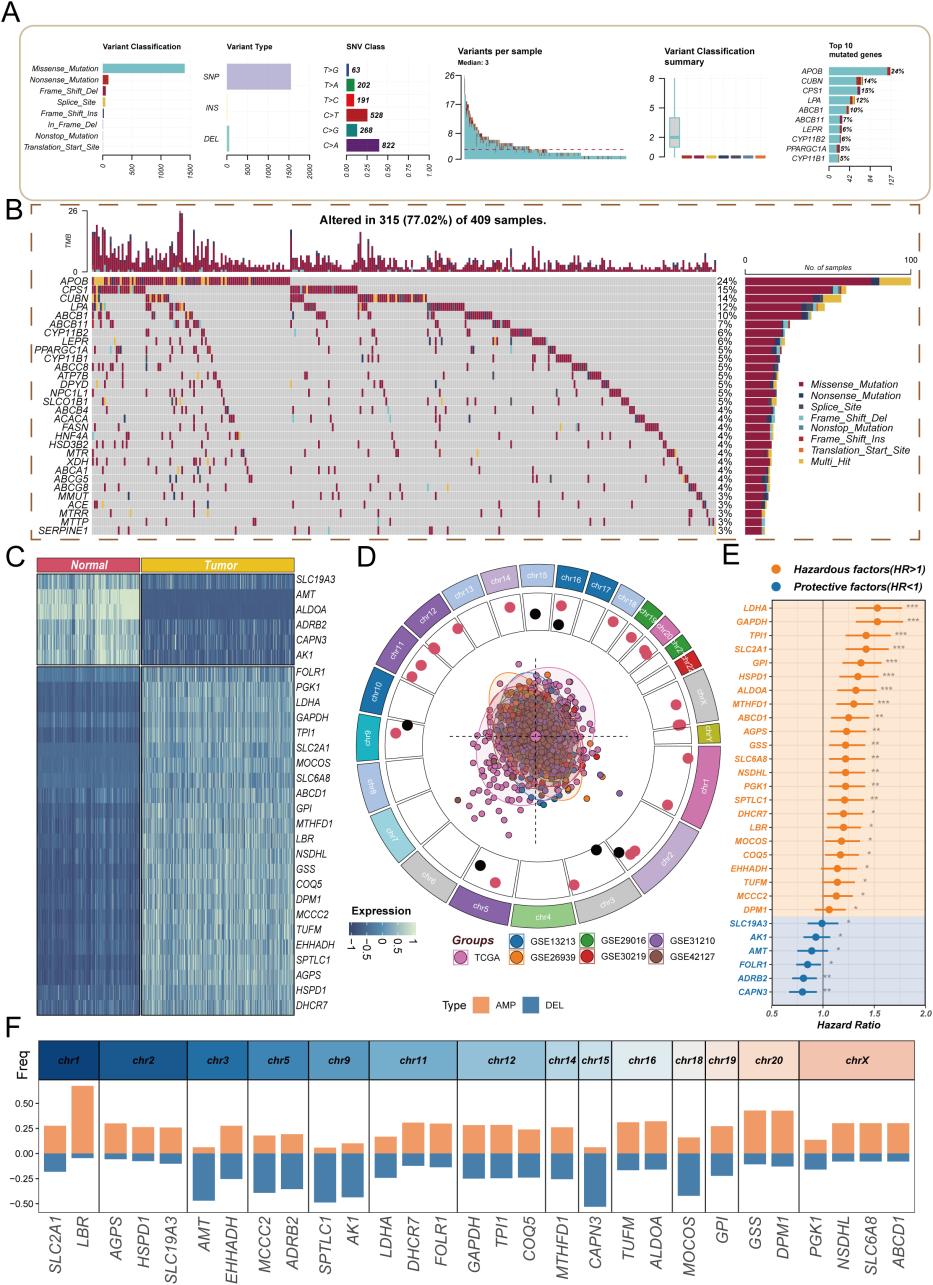
Analysis of molecular characteristics of cholesterol-related genes in lung adenocarcinoma. **(A)** Overview of mutation characteristics, highlighting the predominant mutation types (missense and nonsense mutations) and their relative frequencies, specifically noting the high occurrence of C>A and C>T mutations, with a median mutation load of 3; **(B)** Waterfall plot showing the frequency of gene mutations; **(C)** Heatmap showing differential expression of cholesterol-related genes between tumor and normal samples; **(D)** Chromosomal localization analysis illustrating the distribution of cholesterol-related genes across the genome; **(E)** Forest plot revealing the relationships between cholesterol-related genes and tumor prognosis, indicating potential risk and protective factors; **(F)** Copy number variation analysis highlighting the significant amplification of the LBR gene and the notable deletion of the CAPN3 gene.

In recent years, the role of cholesterol metabolism in the immune microenvironment of LUAD has attracted increasing attention. Based on this, the present study first integrated 1,682 patient samples from 14 cohorts and selected cholesterol metabolism-related genes to construct a CMS, which was used to evaluate prognosis and response to immunotherapy. Subsequently, the CMS was analyzed across multiple cohorts and examined in relation to immune characteristics. Further, multiplex immunofluorescence and *in vitro* and *in vivo* experiments were conducted to investigate the association between the key gene DHCR7 and the immune microenvironment, revealing its involvement in LUAD progression (**Figure 1**). We first explored the mutation characteristics of cholesterol-related genes (**Figures 2A, B**). Analysis revealed that the predominant mutation types were missense mutations and nonsense mutations, with single nucleotide polymorphisms (SNPs) being the most common. Notably, C>A and C>T mutations exhibited higher frequencies. The median mutation load was 3, indicating a significant accumulation of mutations in these genes during tumorigenesis. Among the top 30 mutated genes, APOB, CPS1, and CUBN were ranked highest, with APOB playing a key role in lipid transport, CPS1 involved in amino acid metabolism, and CUBN participating in the absorption and transport of nutrients. Next, we conducted differential analysis of cholesterol-related genes using tumor samples from TCGA and normal samples from GTEx. A COX proportional hazards analysis was performed on seven bulk cohorts, with batch effects removed, leading to the identification of 29 statistically significant genes across at least four datasets for further analysis. The heatmap of differential expression analysis (**Figure 2C**) clearly illustrated the expression differences of these genes between tumor and normal samples, highlighting their importance in the tumor microenvironment. Chromosomal localization analysis (**Figure 2D**) displayed the genomic distribution of these genes. The PCA plot illustrated the sample distribution across the seven bulk cohorts after batch effect removal, providing a basis for subsequent analyses. The forest plot (**Figure 2E**) offered insights into the relationships between these genes and tumor prognosis, revealing potential risk and protective factors, thereby further validating the association between differential genes and clinical outcomes. Finally, an assessment of copy number variation among these genes indicated that LBR exhibited a significant copy number amplification, while CAPN3 showed a notable copy number deletion (**Figure 2F**). These changes may reflect the adaptation of tumor cells to environmental pressures during evolution, emphasizing the critical roles these genes play in tumorigenesis and progression.

### Cholesterol metabolism signature characteristics

**Figure 3:**
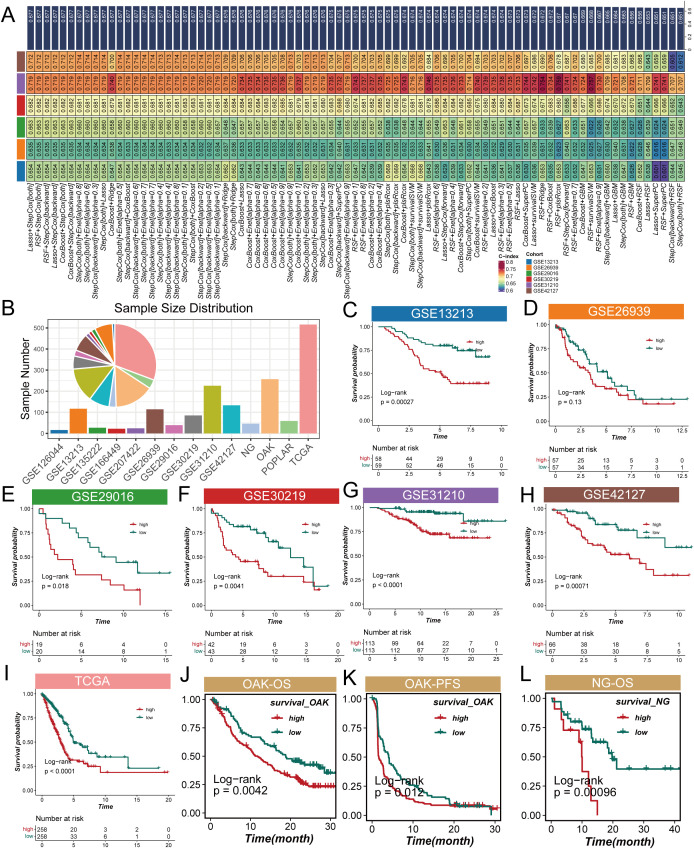
Analysis of cholesterol metabolism signature (CMS) characteristics in lung adenocarcinoma. **(A)** Average concordance index (c-index) of the Lasso + StepCox[both] model across validation datasets, indicating its adaptability and reliability; **(B)** Pie chart demonstrating the proportion of samples from the POPLAR cohort included in the analysis; **(C)** Overall survival (OS) results for the POPLAR cohort; **(D–I)** Survival curves illustrating overall survival (OS) and progression-free survival (PFS) for lung adenocarcinoma patients categorized by low and high CMS levels, with high CMS levels associated with lower survival probabilities; **(J)** Overall survival (OS) analysis for the OAK cohort showing significant differences between high and low CMS groups; **(K)** Progression-free survival (PFS) results for the OAK cohort exhibiting similar trends to overall survival; **(L)** Overall survival (OS) outcomes for the NG cohort further supporting the association between CMS levels and patient prognosis.

To explore the impact of CMS on the prognosis of LUAD patients, this study employed machine learning algorithms using the TCGA dataset as the training group, while selecting six GEO datasets as validation groups to enhance the model’s generalizability. Our calculations revealed that the Lasso + StepCox[both] model achieved the highest average concordance index (c-index) in the validation groups (**Figure 3A**), indicating its strong adaptability and reliability across different datasets. After establishing the model, we analyzed the sample sizes across the datasets. The pie chart in **Figure 3B** visually demonstrates the proportion of each dataset in the sample, providing a foundation for interpreting subsequent results. Next, we assessed the role of CMS in the survival of LUAD patients. The survival curves presented in **Figures 3C–I** indicate that the survival probability of the high CMS group is significantly lower than that of the low CMS group. This directly supports the importance of cholesterol metabolism characteristics in evaluating patient prognosis, showing that high CMS levels are associated with poorer survival outcomes. Specifically, **Figures 3J, L** illustrate overall survival (OS) results, while **Figure 3K** presents progression-free survival (PFS) results, with no significant differences observed in the remaining corresponding PFS and OS results (see **Supplementary Figures 1A-C**). We then further explored the differences in CMS between the responder and non-responder groups across four NSCLC cohorts. The results indicate that consistently low CMS values appear to correlate with better immunotherapy outcomes (see **Supplementary Figures 1D-G**). These findings emphasize that CMS can serve as a stable biomarker to predict the prognosis and immunotherapy efficacy in LUAD patients.

### Comprehensive validation of cholesterol metabolism signature

**Figure 4:**
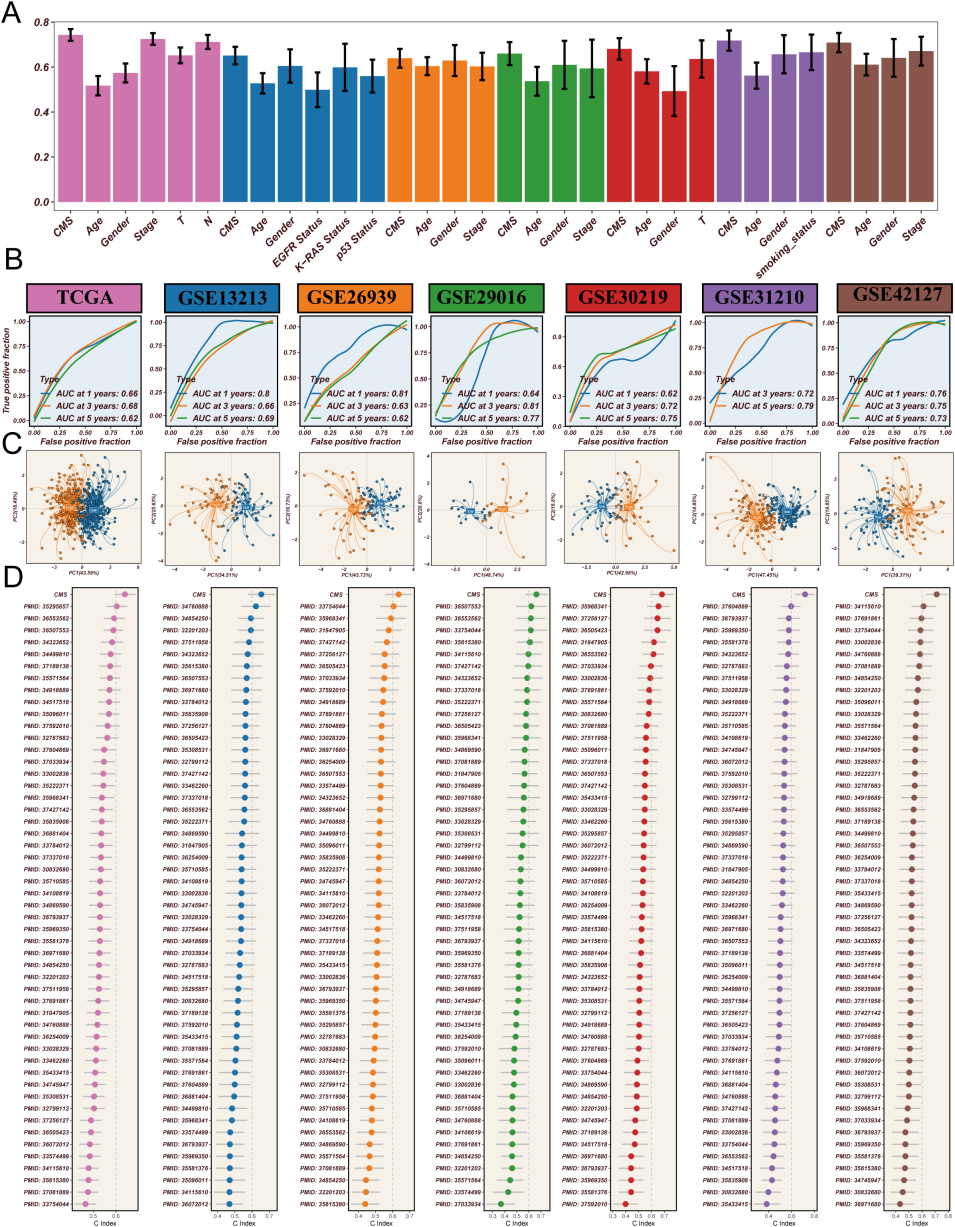
Comprehensive validation of cholesterol metabolism signature (CMS) in lung adenocarcinoma prognosis. **(A)** Comparison of c-index values between CMS and traditional clinical indicators, demonstrating the superior prognostic predictive power of CMS across different clinical features, with CMS consistently showing the highest predictive performance; **(B)** ROC curve analysis across multiple cohorts, showing AUC values consistently above 0.65 in the training set (TCGA cohort) and validation sets (GSE13213, GSE26939, GSE29016, GSE30219, GSE31210, GSE42127), indicating robust prognostic prediction capabilities across different datasets; **(C)** Principal Component Analysis (PCA) based on CMS feature genes, revealing distinct molecular-level clustering of samples and clearly differentiating patient populations with different risk profiles; **(D)** Comparative analysis against 51 previously published prognostic signatures, highlighting CMS’s superior performance with the highest c-index values across all validation cohorts, demonstrating its potential as a novel prognostic marker for lung adenocarcinoma.

To systematically evaluate the prognostic predictive performance of the CMS in LUAD, we conducted a comprehensive validation analysis. **Figure 4A** illustrates the comparison of CMS with traditional clinical indicators in prognostic prediction. The results revealed that traditional clinical features such as age, gender, and tumor stage showed relatively low c-index values, while CMS demonstrated significantly higher predictive power. Specifically, the c-index values were 0.58 for age, 0.55 for gender, and 0.62 for tumor stage, in contrast to CMS reaching 0.83, indicating its potential superiority as a prognostic marker. ROC curve analysis (**Figure 4B**) assessed the predictive performance of CMS. In both training and validation sets, the AUC values consistently exceeded 0.65, demonstrating the model’s robust prognostic prediction capability. The stable performance across different cohorts provides strong support for CMS as a prognostic prediction tool for LUAD. To explore CMS’s molecular-level discrimination ability, we performed Principal Component Analysis (PCA) (**Figure 4C**). The results revealed distinct spatial distribution patterns between high-score and low-score samples across all validation cohorts based on CMS feature genes. This clustering characteristic suggests that CMS is not merely a statistical indicator but can effectively distinguish patient populations with different prognostic risks at the molecular level. Finally, we conducted a rigorous benchmark test for CMS. By systematically comparing 51 previously published LUAD prognostic signatures (**Figure 4D**), we found that CMS consistently maintained the highest c-index values across all validation cohorts, significantly outperforming all previously published prognostic models. In conclusion, through multi-angle and multi-dimensional analyses, we comprehensively validated the excellent prognostic predictive performance of the CMS. Whether compared with clinical indicators, ROC curve analysis, molecular-level validation, or benchmarking against existing models, CMS demonstrated remarkable advantages. This not only provides a new molecular strategy for personalized prognosis assessment of LUAD but also offers a new research perspective for understanding tumor progression mechanisms, particularly the potential key role of cholesterol metabolism in tumor biology.

### Cholesterol metabolism signature shapes immune microenvironment

**Figure 5:**
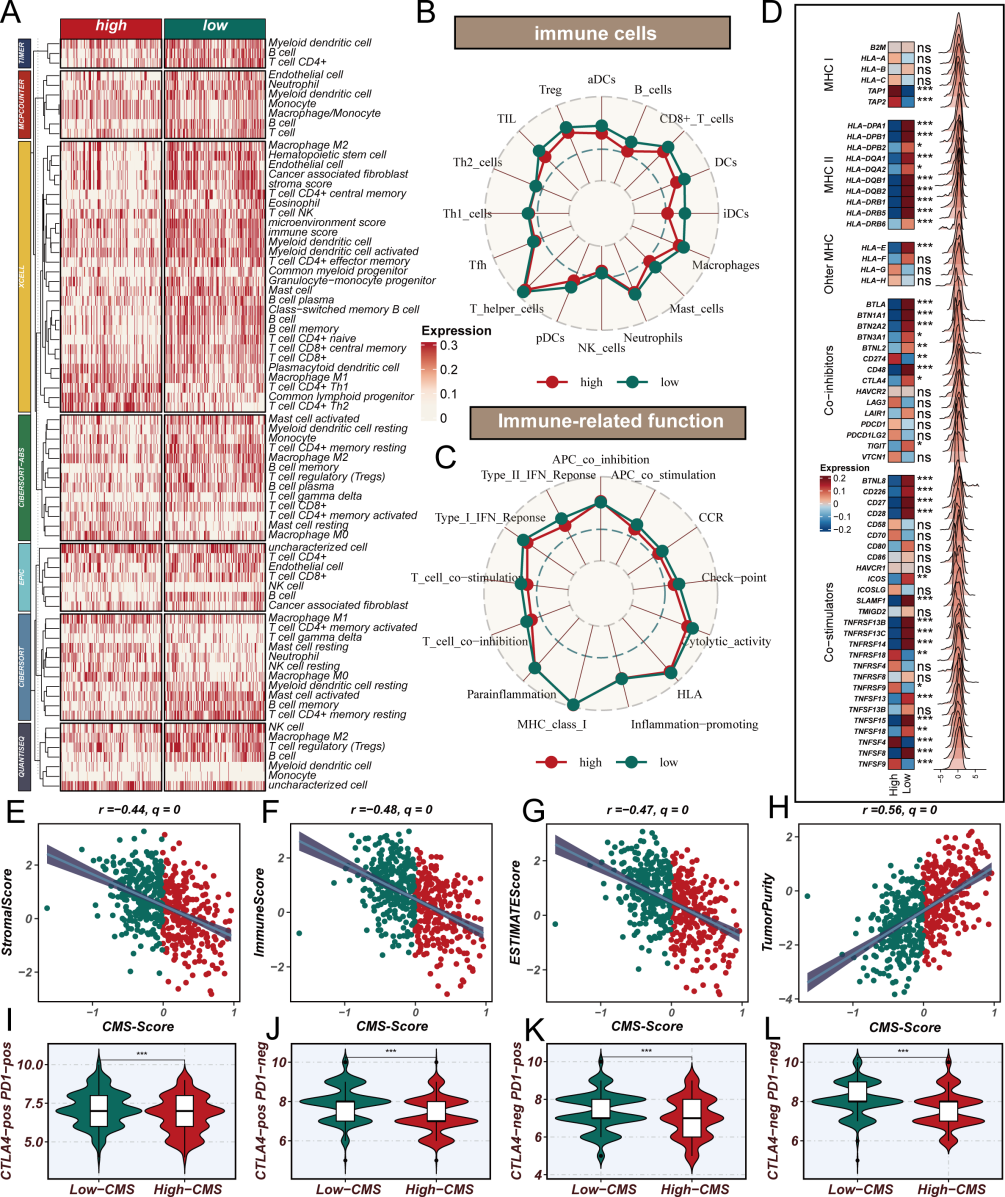
Cholesterol Metabolism Signature Shapes Immune Microenvironment in Lung Adenocarcinoma. **(A)** Immune cell infiltration analysis using TIMER 2.0 platform across seven different algorithms, revealing significantly higher infiltration of CD8+ T cells, B cells, dendritic cells (DCs), and natural killer (NK) cells in the low CMS group compared to the high CMS group; **(B, C)** ssGSEA algorithm analysis of immune cell infiltration and functional characteristics, showing detailed visualization of immune cell populations and immune-related functions, highlighting the more active immune profile of the low CMS group; **(D)** Differential expression of immune-related genes, focusing on MHC II antigen gene expression, with significantly upregulated MHC II class antigen genes in the low CMS group, suggesting enhanced antigen presentation capabilities; **(E–H)** ESTIMATE algorithm assessment of immune microenvironment features, demonstrating negative correlation between CMS and immune scores, positive correlation with tumor purity, and comprehensive mapping of immune activity variations; **(I–L)** TCIA Immunotherapy Potential Score (IPS) analysis, revealing significantly higher potential for immunotherapeutic response in the low CMS group.

To comprehensively investigate the impact of CMS on the immune microenvironment in LUAD, we conducted an extensive bioinformatics analysis. The influence of cholesterol metabolism characteristics on the immune microenvironment in LUAD represents a complex, multidimensional process. Initially, using the TIMER 2.0 platform to analyze TCGA database with seven different algorithms for immune infiltration (**Figure 5A**), we observed that the low CMS group demonstrated significantly higher infiltration of multiple immune cell types. Specifically, the low CMS group exhibited elevated levels of CD8+ T cells, B cells, dendritic cells (DCs), and natural killer (NK) cells. To further elucidate the precise changes in immune cells, we employed ssGSEA algorithm to analyze immune cell infiltration and function (**Figures 5B, C**). The results revealed that the low CMS group displayed markedly more active immune cell infiltration and functional characteristics. Immunology-related gene analysis (**Figure 5D**) uncovered deeper mechanistic insights. The low CMS group showed significantly upregulated MHC II antigen gene expression, suggesting enhanced antigen presentation capabilities that could promote T cell recognition and activation. MHC II molecules play a crucial role in tumor immune surveillance, and their high expression may be a key reason for the more robust immune activity in the low CMS group. Through ESTIMATE algorithm assessment of immune microenvironment features (**Figures 5E–H**), we discovered a negative correlation between CMS and immune scores, while observing a positive correlation with tumor purity. This indicates that as CMS scores increase, tumor immune activity gradually decreases, accompanied by a proportional increase in tumor cell content. This finding suggests that CMS not only reflects tumor metabolic characteristics but may also directly influence tumor immune escape mechanisms. Lastly, the TCIA Immunotherapy Potential Score (IPS) analysis (**Figures 5I–L**) further substantiated our hypothesis. The significantly higher IPS scores in the low CMS group suggest that these patients may exhibit better responses to immunotherapy, providing crucial molecular stratification for personalized immune interventions. In conclusion, our research reveals the intricate associations between cholesterol metabolism characteristics and the tumor immune microenvironment. The low CMS group demonstrates a more active immune microenvironment, potentially attributed to its superior antigen presentation capabilities and immune cell infiltration characteristics.

### CMS-mediated immune suppression

**Figure 6:**
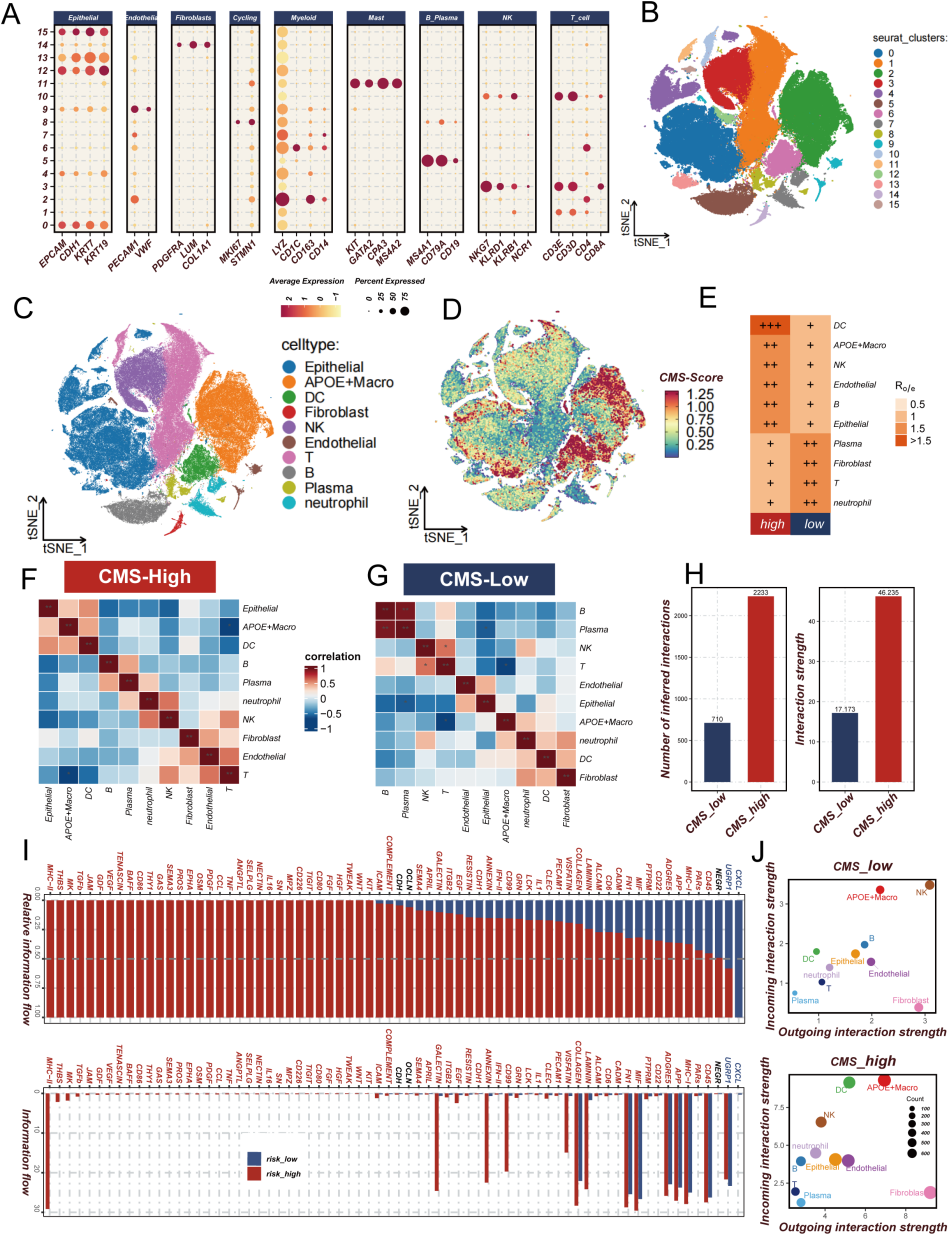
Analysis of cholesterol metabolism signature (CMS) characteristics in lung adenocarcinoma. **(A, B)** Expression and t-SNE distribution of characteristic marker genes in different cell clusters; **(C)** Annotation of 10 cell types in lung adenocarcinoma microenvironment; **(D)** CMS score distribution across different cell types, highlighting concentrated expression in APOE macrophages and dendritic cells; **(E)** Ro/e analysis comparing cellular composition between high and low CMS groups, showing enrichment patterns of specific cell populations; **(F, G)** Cell co-occurrence analysis in high and low CMS environments; **(H)** Comparison of intercellular communication intensity and quantity between low and high CMS groups; **(I)** Signal pathway analysis revealing immune regulatory pathway activation in high and low CMS groups; **(J)** Comparative diagram of cellular signal intensity in and out of low and high CMS groups. * P<0.05.

In the context of our previous investigation into the CMS and its immunological implications in tumor microenvironments, single-cell transcriptomic analysis was performed to elucidate the underlying cellular mechanisms. Unsupervised clustering was initially conducted on the single-cell dataset, resulting in the identification of 16 distinct cellular clusters (**Figure 6B**). Cell type annotation was achieved through comprehensive marker gene expression profiling (**Figure 6A**), subsequently categorizing cells into 10 distinct populations (**Figure 6C**). The proportion of cell type abundance in each sample was **Supplementary Figure 2A**. A CMS scoring algorithm was implemented to quantify the molecular signature, with t-SNE visualization revealing concentrated expression patterns (**Figure 6D**). Notably, elevated CMS scores were predominantly observed in APOE-expressing macrophages and dendritic cells, with significant epithelial cell representation. Comparative analysis between high and low CMS groups demonstrated differential cellular composition. The high CMS group was characterized by significant enrichment of macrophages, dendritic cells, and epithelial cells, in contrast to the low CMS group, which was predominantly populated by T cells and plasma cells (**Figure 6E**). Cellular co-occurrence analysis (**Figures 6F, G**) revealed distinctive microenvironmental characteristics. Enhanced intercellular communication was documented in the high CMS group, with increased signaling complexity and interaction intensity (**Figure 6H**; **Supplementary Figure 2B**). Pathway enrichment analysis disclosed upregulation of immunomodulatory signaling cascades, including PDGF and MK pathways, which have been previously associated with immunosuppressive microenvironmental configurations (**Figure 6I**). Mechanistic interrogation of cellular signaling dynamics demonstrated heightened signal transduction capabilities of APOE-positive macrophages within the high CMS cohort (**Figure 6J**). These observations align with established immunological literature documenting the immunosuppressive potential of APOE-expressing macrophage populations in neoplastic environments. Collectively, this single-cell resolution investigation provides unprecedented insights into the intricate role of CMS in tumor microenvironment remodeling. A complex interplay between APOE-positive macrophages, dendritic cells, and epithelial cells was characterized, revealing mechanisms underlying immunosuppressive microenvironmental programming.

### DHCR7-mediated immunosuppression

**Figure 7:**
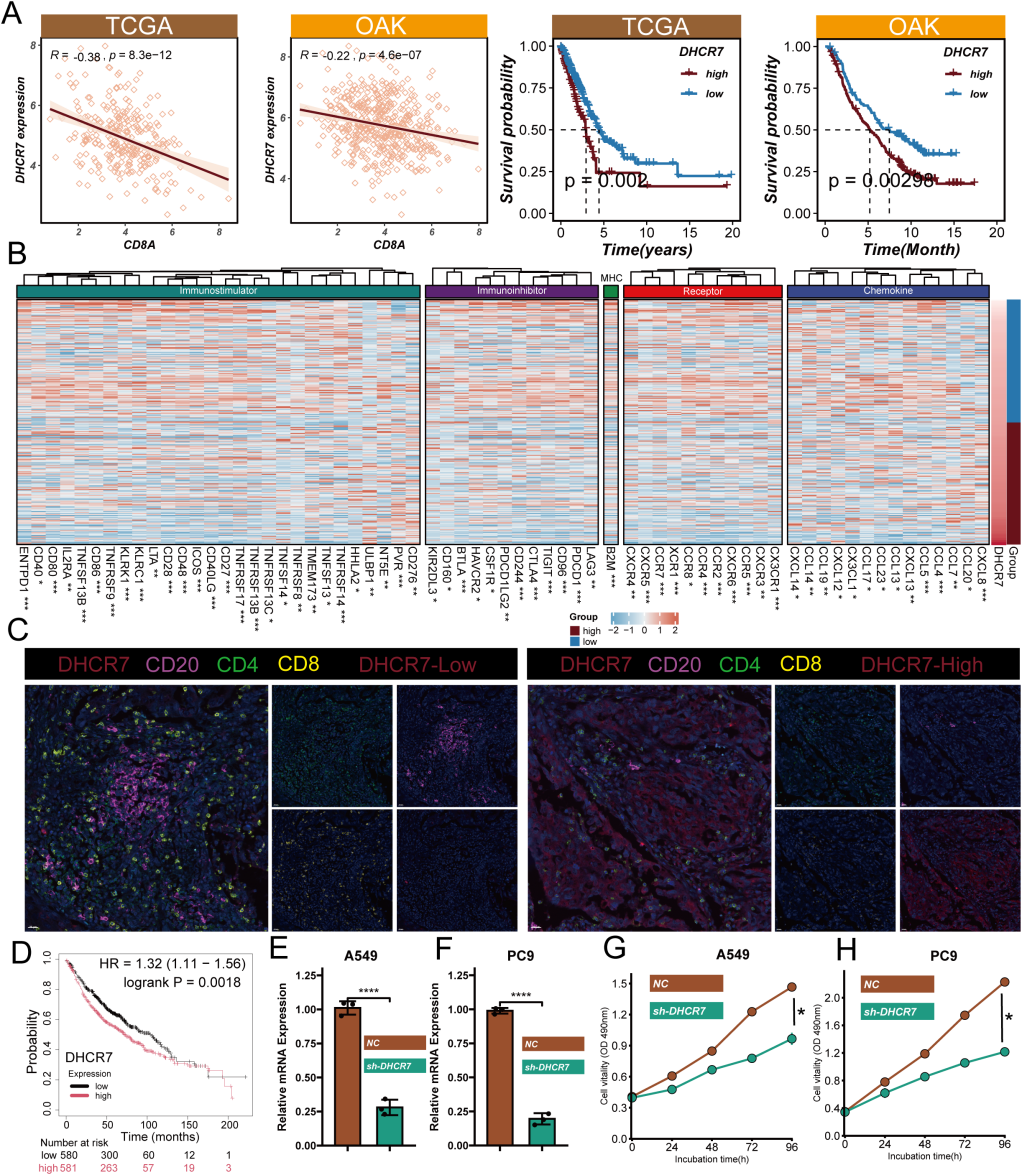
Functional Validation and Mechanistic Insights of DHCR7 in Lung Adenocarcinoma. **(A)** Gene correlation of DHCR7 and CD8A and DHCR7 prognostic analysis in TCGA and OAK cohorts; **(B)** Heatmap of immune-related gene expression correlated with DHCR7 levels in OAK data; **(C)** Multiplex immunofluorescence image showing immune cell distribution in high and low DHCR7 expression regions; **(D)** The KM-plotter website further confirmed that LUAD patients with high DHCR7 expression have a poorer prognosis; **(E, F)** Verification of DHCR7 gene knockdown efficiency in A549 and PC9 cell lines using qRT-PCR and Western blot; **(G, H)** Cell proliferation assays (CCK8) showing significant growth inhibition after DHCR7 knockdown. * P<0.05; ** P<0.01; *** P<0.001; **** P<0.0001.

Interestingly, we discovered that DHCR7, as a key model gene, shows a significant positive correlation with CMS scores (r=0.42, q=0) (**Supplementary Figure 3A**). This finding reveals the potential important role of DHCR7 in LUAD molecular progression. Immunohistochemical staining from the Human Protein Atlas (HPA) database demonstrated that DHCR7 expression was significantly upregulated in LUAD tissues compared to normal lung tissues, with staining intensity shifting from low intensity (<25%) to high intensity (>75%), localized in the cytoplasm and cell membrane (**Supplementary Figure 3B**). To verify the immune-related function of DHCR7, we integrated TCGA and OAK immunotherapy cohorts for in-depth analysis. Results showed a significant negative correlation between DHCR7 and the key immune-related gene CD8A (TCGA cohort: R=-0.38, p=8.3e-12; OAK cohort: R=-0.22, p=4.6e-07) (**Figure 7A**). Survival analysis revealed that patients with high DHCR7 expression had significantly shorter overall and progression-free survival compared to the low expression group (p<0.001). Additionally, validation using the KM Plotter database confirmed that higher DHCR7 expression is significantly associated with poorer survival outcomes, further supporting our survival analysis findings (**Figure 7D**). Surprisingly, we found that as DHCR7 expression increased, key immune regulatory genes, including immunostimulatory factors, immunosuppressive factors, MHC molecules, cytokine receptors, and chemokines, showed a progressive downregulation, which was observed in both OAK and TCGA cohorts (**Figure 7B**, **Supplementary Figure 4A**). Furthermore, we discovered that DHCR7 displayed a clear negative correlation with critical immune circulation steps (CD8T recruitment), while showing a positive correlation with cell cycle and DNA replication pro-oncogenic pathways (**Supplementary Figure 4B**). Multiplex immunohistochemical images clearly demonstrated that CD20+, CD4+, and CD8+ T cell infiltration was significantly higher in DHCR7 low-expression areas compared to high-expression areas (**Figure 7C**), further confirming our previous findings. To validate our functional hypothesis, we performed DHCR7 gene knockdown experiments in A549 and PC9 LUAD cell lines. Cell proliferation assays (CCK8) showed that DHCR7 knockdown significantly reduced cell proliferation capacity, resulting in a flattened growth curve (**Figures 7E–H**). In summary, we discovered that DHCR7 is not only a core gene in the CMS model but may also participate in LUAD progression by regulating the immune microenvironment and cell proliferation processes.

### DHCR7 regulates LUAD progression

**Figure 8:**
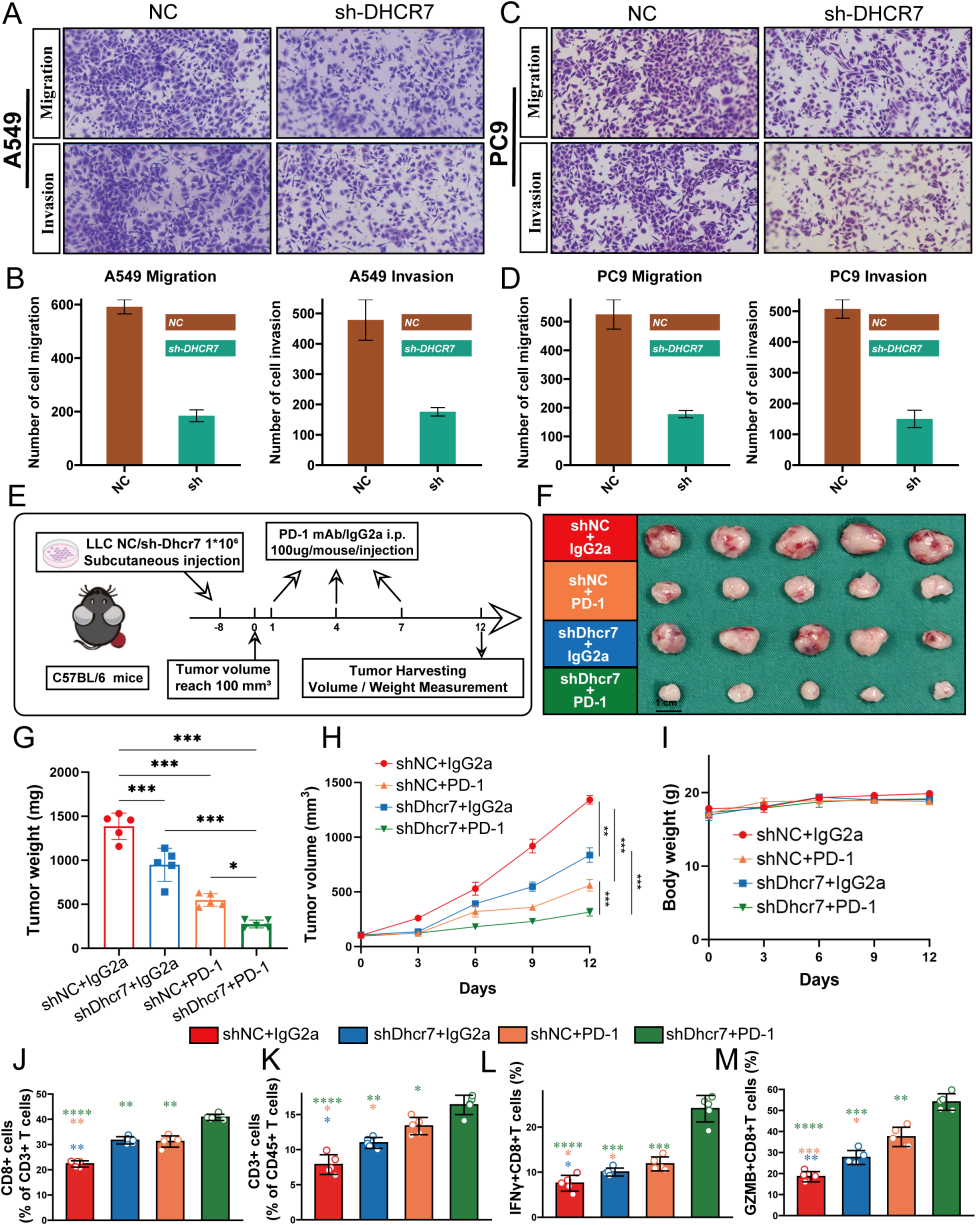
DHCR7 Regulates Lung Adenocarcinoma Progression through Multiple Mechanisms. **(A–D)** Migration and invasion assays reveal significant inhibition of A549 and PC9 cell motility and invasiveness upon DHCR7 knockdown. **(E)** Schematic illustration of the mouse xenograft tumor model experimental design. **(F–H)** Tumor growth analyses demonstrate reduced tumor volume and weight across different experimental groups, with notable suppression in shDhcr7+IgG2a and shDhcr7+PD-1 groups. **(I)** Mouse body weight changes monitored throughout the experiment. **(J–M)** Flow cytometry analysis unveils substantial alterations in immune cell subsets, including increased proportions of CD8+ T cells, CD3+ T cells, IFN-γ+ T cells, and GZMB+ CD8+ T cells, highlighting DHCR7's profound impact on the tumor immune microenvironment. * P<0.05; ** P<0.01; *** P<0.001; **** P<0.0001.

High DHCR7 expression was associated with consistently worse survival across seven lung adenocarcinoma datasets (**Supplementary Figure 5**). To further verify the biological function of DHCR7, we first explored its role in LUAD cell migration and invasion through *in vitro* experiments. Migration and invasion assay results showed that DHCR7 gene knockdown significantly inhibited the migration and invasion capabilities of A549 and PC9 LUAD cell lines compared to the control group (NC) (**Figures 8A–D**). Quantitative analysis revealed that cell migration and invasion numbers decreased by approximately 60-70% (**Figures 8B, D**), indicating that DHCR7 may play a critical role in tumor metastasis. To further validate DHCR7’s biological function in tumor growth, we constructed a mouse xenograft tumor model (**Figure 8E**). The *in vivo* experimental results were highly consistent with the *in vitro* research findings. We established four experimental groups: shNC+IgG2a (control group), shNC+PD-1, shDhcr7+IgG2a, and shDhcr7+PD-1. Compared to the control group, the DHCR7 knockdown group (shDhcr7+IgG2a) showed significantly inhibited tumor growth, with markedly reduced tumor volume and weight (**Figures 8F–H**). During the 12-day observation period, the tumor volume growth rate of the DHCR7 knockdown group was significantly slowed. Notably, the DHCR7 knockdown group combined with PD-1 inhibition (shDhcr7+PD-1) demonstrated more significant tumor growth suppression, with further reduced tumor volume and weight compared to the group with DHCR7 knockdown alone. The body weight changes across all groups remained stable (**Figure 8I**), indicating that the experimental treatment did not significantly affect the overall health of the animals. To deeply explore DHCR7’s impact on the tumor immune microenvironment, we performed a detailed analysis of immune cell subsets in tumor tissues using flow cytometry. The results showed significant changes in immune cell distribution in the DHCR7 knockdown group compared to the control group. Specifically, the proportions of CD8+ T cells (**Figure 8J**), CD3+ T cells (**Figure 8K**), IFN-γ+ T cells (**Figure 8L**), and GZMB+ CD8+ T cells (**Figure 8M**) were significantly increased. This finding suggests that DHCR7 may influence tumor progression by regulating immune cell infiltration. Notably, the DHCR7 knockdown group combined with PD-1 inhibition (shDhcr7+PD-1) showed further enhanced immune cell activity, implying a potential synergistic effect between DHCR7 and PD-1. In conclusion, DHCR7 demonstrates multiple regulatory functions in LUAD development, providing important theoretical basis for DHCR7 as a potential therapeutic target.

## Discussion

As the most common subtype of lung cancer, the complexity of LUAD’s occurrence and development has long been a major challenge in tumor research (42–44). With the rapid development of tumor immunology and precision medicine, the critical role of metabolic reprogramming in tumor biology has gradually become a research hotspot (45, 46). Cholesterol metabolism, as an essential life process of cells, has attracted significant attention for its regulatory mechanisms in tumor progression (47). By integrating 1,682 patient samples from 14 cohorts, this study aims to comprehensively reveal the molecular mechanisms and immune regulatory effects of cholesterol metabolism in LUAD development.

Gene mutations have been a core focus of tumor research as key drivers of tumor occurrence and development (48, 49). Our systematic analysis of cholesterol-related gene mutations revealed that these genes primarily undergo missense and nonsense mutations, predominantly single nucleotide polymorphisms (SNPs). The high incidence of C>A and C>T mutation types, with a median mutation burden of 3, indicates significant genetic variations in these genes during tumor progression. The heterogeneity and individual differences of tumors have always been major challenges for precision medicine (50, 51). Traditional clinical prognosis assessment methods are often limited to tumor staging, age, and mutations, making it difficult to comprehensively reflect the complex biological characteristics of tumors. In recent years, prognosis models based on molecular characteristics have become a focal point of precision oncology research. We innovatively constructed a CMS model to provide a more precise prognostic assessment tool. Using advanced machine learning algorithms, particularly the Lasso + StepCox[both] model, we demonstrated excellent predictive performance across multiple independent validation cohorts, significantly surpassing traditional clinical indicators. Compared to single traditional indicators, the CMS model not only improved prognostic prediction accuracy but also opened up new research directions for personalized precise treatment of LUAD.

The immune microenvironment plays a crucial role in tumor progression and has become a cutting-edge field in tumor immunology research (52, 53). Previous studies primarily focused on tumor immune escape mechanisms, with relatively limited research on the interactive regulation between metabolic pathways and the immune microenvironment (54). Through single-cell transcriptome analysis, we discovered that the low CMS group exhibits more active immune cell infiltration and functional characteristics. The low CMS group showed significant increases in CD8+ T cells, B cells, dendritic cells, and natural killer (NK) cells, with upregulated MHC II antigen gene expression, suggesting enhanced antigen presentation capabilities. This finding provides a new perspective for understanding the regulatory mechanisms of the tumor immune microenvironment. DHCR7, as a key gene in the CMS model, was comprehensively validated through *in vitro* and *in vivo* experiments for its multidimensional regulatory effects in LUAD progression. The research found that DHCR7 is highly expressed in LUAD tissues and shows a significant negative correlation with key immune-related genes (such as CD8A). Gene knockdown experiments demonstrated not only significant inhibition of tumor growth but also enhanced immune cell infiltration, particularly with notable increases in IFN-γ+ CD8+ T cells and GZMB+ CD8+ T cell proportions. This finding provides strong experimental evidence for DHCR7 as a potential therapeutic target.

This study constructed a multi-dimensional regulatory model from molecules to cells, from genes to immunity, providing a new theoretical framework for understanding LUAD development. Despite significant progress, the study has limitations. Future research requires larger-scale prospective clinical studies to further validate our findings and explore the precise mechanism of DHCR7 in regulating the immune microenvironment.

In conclusion, by constructing a CMS model centered on DHCR7, this study systematically elucidates the multidimensional regulatory mechanisms of cholesterol metabolism in LUAD development, providing important clues for personalized immunotherapy strategies and pointing out new directions for precise LUAD treatment research.

## Data Availability

The original contributions presented in the study are included in the article/**Supplementary Material**. Further inquiries can be directed to the corresponding authors.

## Supplementary material

Supplementary Figure 1 Additional Analysis of CMS and Treatment Response in Lung Cancer Cohorts. **(A)** Progression-free survival (PFS) results for the NG cohort confirming the correlation between CMS levels and patient prognosis; **(B)** Overall survival (OS) results for the POPLAR cohort; **(C)** Progression-free survival (PFS) results for the POPLAR cohort; **(D–G)** Comparative analysis between responder and non-responder groups across four non-small cell lung cancer cohorts, indicating that consistently low CMS values are associated with improved immunotherapy responses.

Supplementary Figure 2 Extended Analysis of Single-Cell Transcriptomics in Lung Adenocarcinoma. **(A)** Cellular type abundance proportion analysis across samples, revealing distribution of 10 major cell populations; **(B)** Comparative analysis of intercellular communication intensity between high and low CMS groups, demonstrating differences in signal transmission and interaction complexity.

Supplementary Figure 3 Expression Characteristics and Correlation Analysis of DHCR7 in Lung Adenocarcinoma. **(A)** Correlation analysis between DHCR7 expression and CMS scores, demonstrating a significant positive correlation (r=0.42, q=0); **(B)** Immunohistochemical staining from the Human Protein Atlas (HPA) database, comparing DHCR7 expression patterns in normal lung and lung adenocarcinoma tissues, visually displaying changes in staining intensity and cellular localization.

Supplementary Figure 4 Molecular Pathway and Immune Correlation Analysis of DHCR7. **(A)** Heatmap of immune-related gene expression correlated with DHCR7 levels in TCGA data, showing progressive downregulation of key immune regulatory genes; **(B)** Network analysis of DHCR7’s association with cellular processes, demonstrating its negative correlation with immune cell recruitment and positive correlation with cell cycle and DNA replication pathways.

Supplementary Figure 5 Prognostic significance of DHCR7 across seven lung adenocarcinoma cohorts.
