# Supplementary figures and images for: Cholesterol metabolism shapes immune low-response states in LUAD: a multi-omics cholesterol metabolism signature predicts immunotherapy benefit and identifies DHCR7 as a therapeutic target
Source: Front Immunol. 2025 Oct 30;16:1696360. doi: 10.3389/fimmu.2025.1696360 (PMC12611822; doi:10.3389/fimmu.2025.1696360)

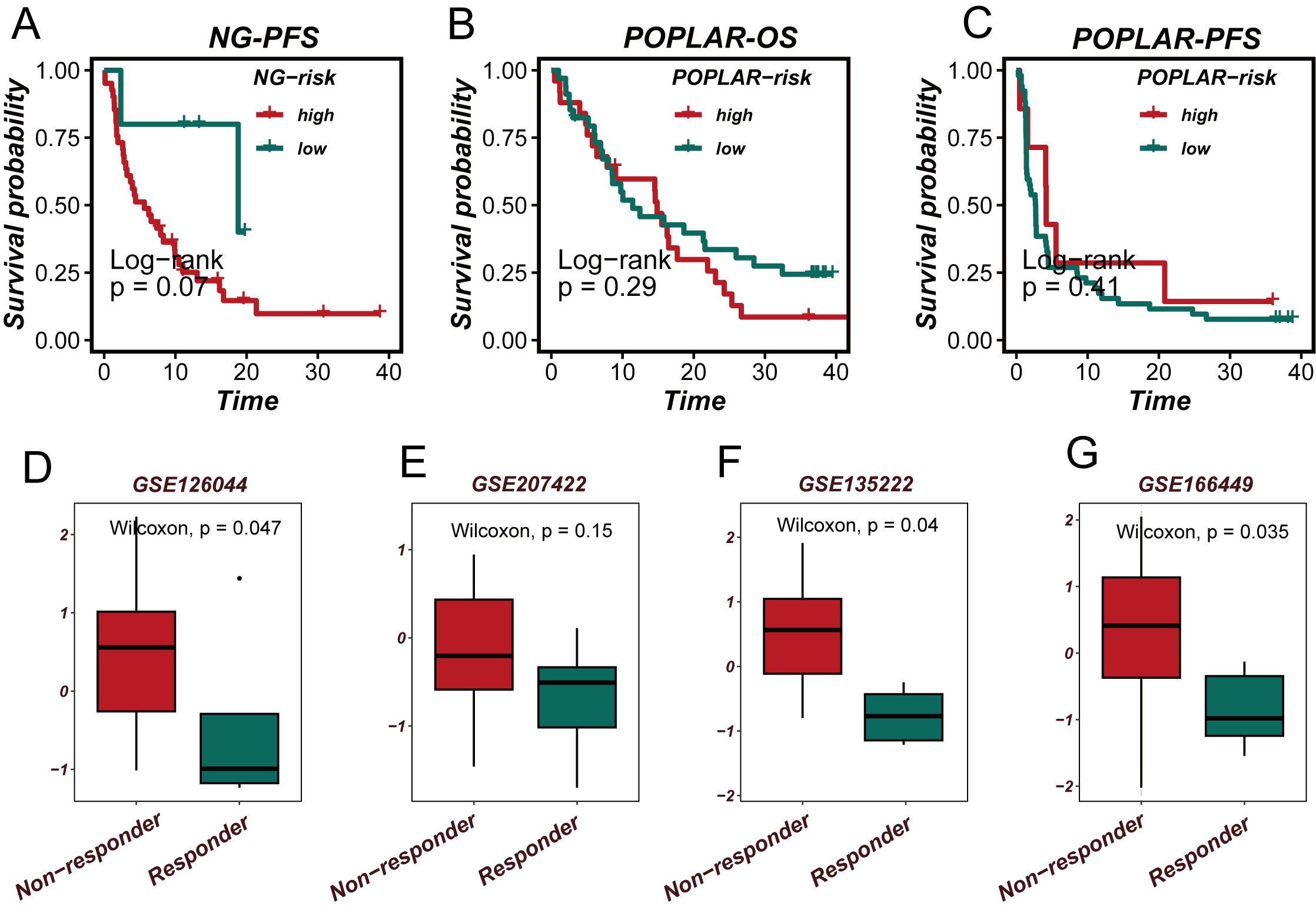

Supplement: Supplementary Figure 1 — Additional Analysis of CMS and Treatment Response in Lung Cancer Cohorts. (A) Progression-free survival (PFS) results for the NG cohort confirming the correlation between CMS levels and patient prognosis; (B) Overall survival (OS) results for the POPLAR cohort; (C) Progression-free survival (PFS) results for the POPLAR cohort; (D–G) Comparative analysis between responder and non-responder groups across four non-small cell lung cancer cohorts, indicating that consistently low CMS values are associated with improved immunotherapy responses. [file Image1.tif]

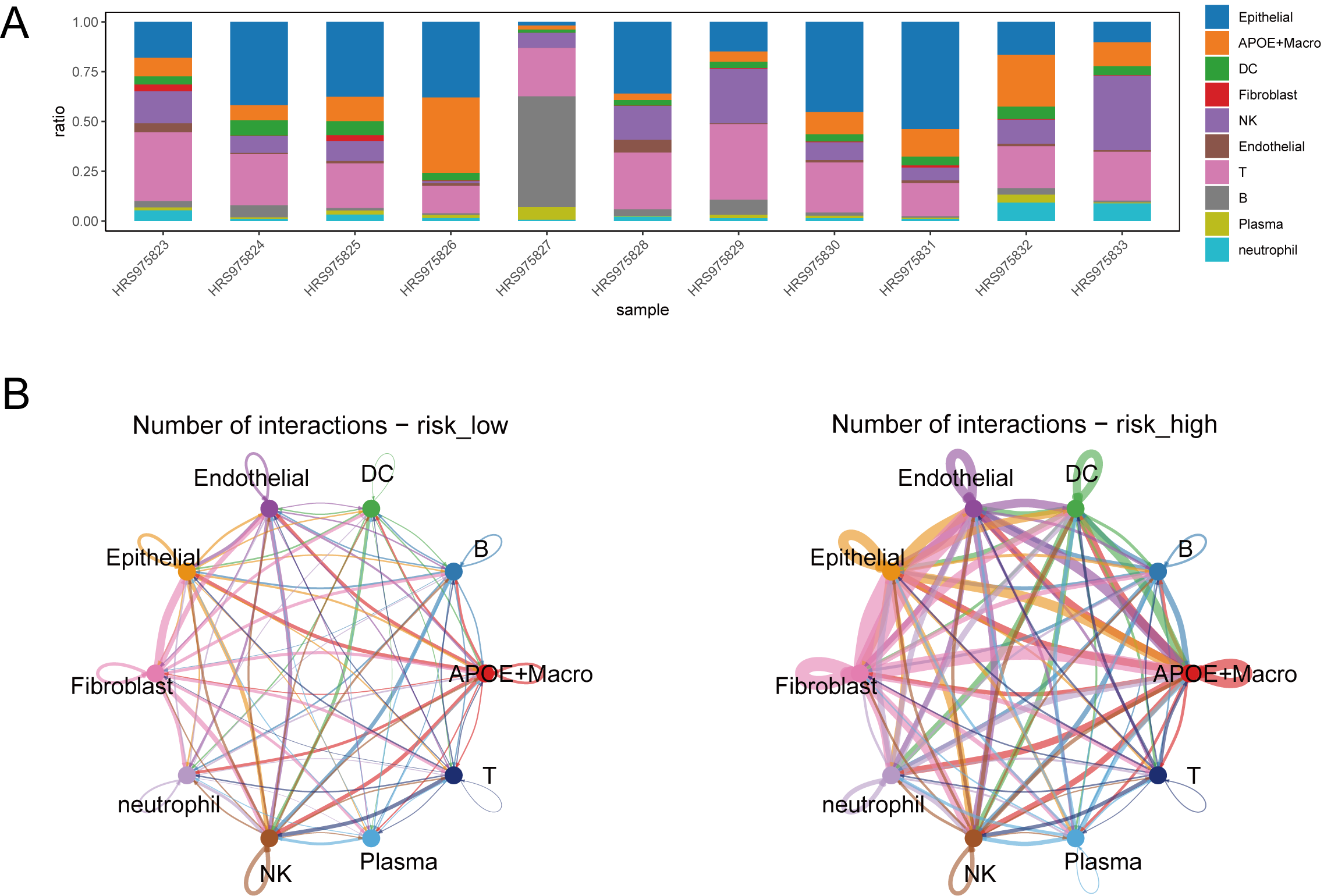

Supplement: Supplementary Figure 2 — Extended Analysis of Single-Cell Transcriptomics in Lung Adenocarcinoma. (A) Cellular type abundance proportion analysis across samples, revealing distribution of 10 major cell populations; (B) Comparative analysis of intercellular communication intensity between high and low CMS groups, demonstrating differences in signal transmission and interaction complexity. [file Image2.tif]

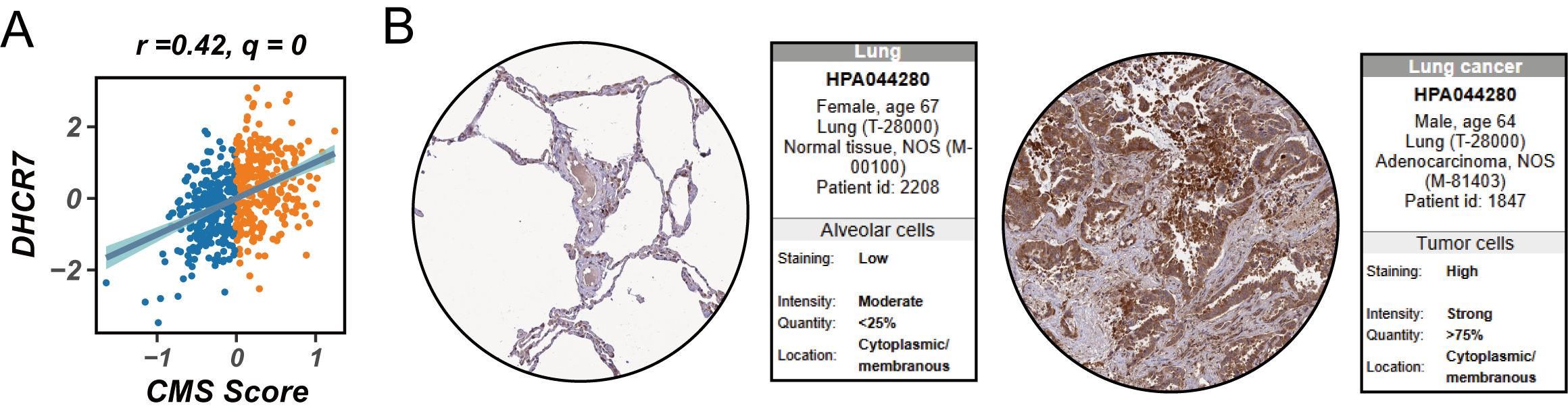

Supplement: Supplementary Figure 3 — Expression Characteristics and Correlation Analysis of DHCR7 in Lung Adenocarcinoma. (A) Correlation analysis between DHCR7 expression and CMS scores, demonstrating a significant positive correlation (r=0.42, q=0); (B) Immunohistochemical staining from the Human Protein Atlas (HPA) database, comparing DHCR7 expression patterns in normal lung and lung adenocarcinoma tissues, visually displaying changes in staining intensity and cellular localization. [file Image3.tif]

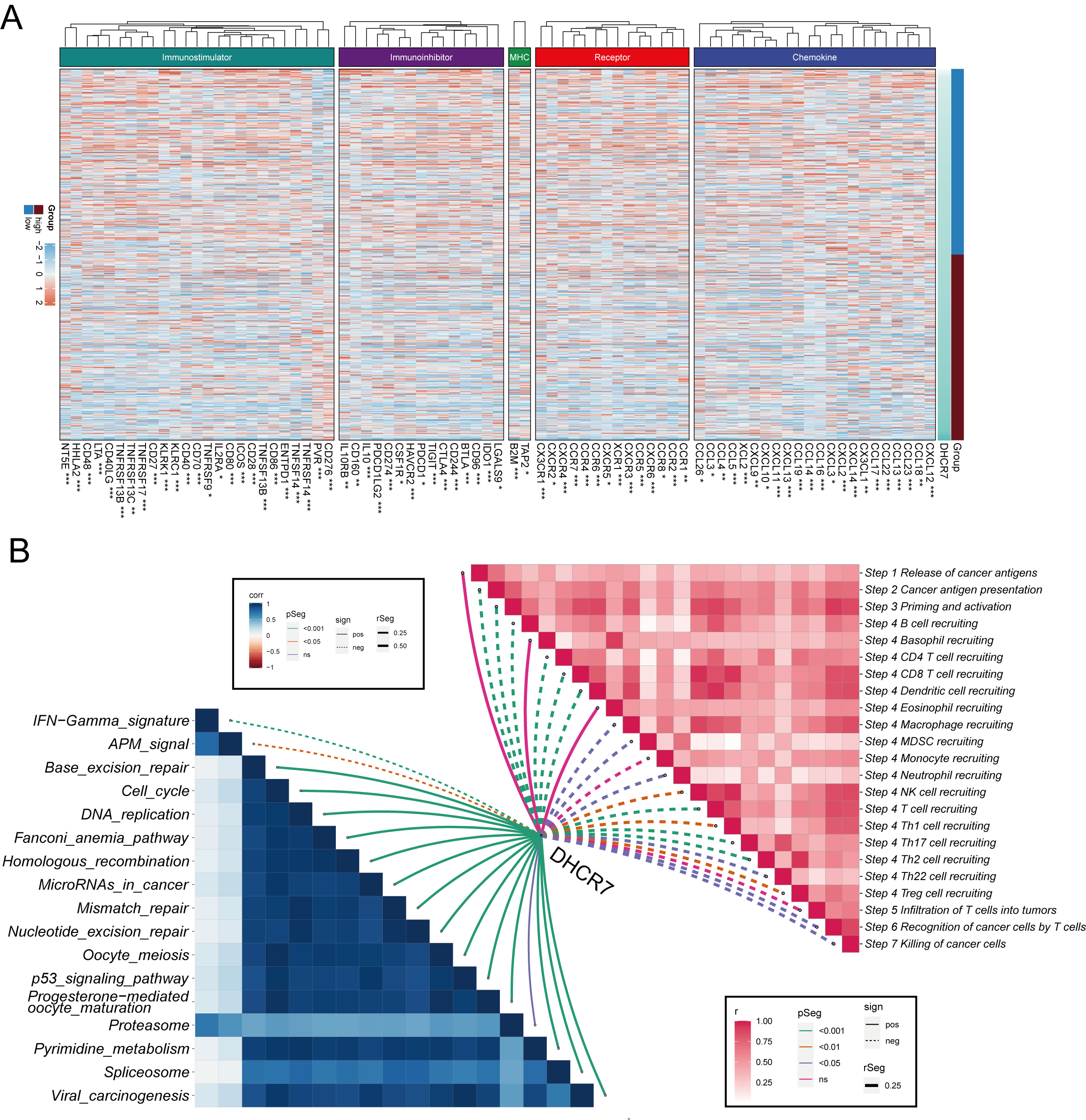

Supplement: Supplementary Figure 4 — Molecular Pathway and Immune Correlation Analysis of DHCR7. (A) Heatmap of immune-related gene expression correlated with DHCR7 levels in TCGA data, showing progressive downregulation of key immune regulatory genes; (B) Network analysis of DHCR7’s association with cellular processes, demonstrating its negative correlation with immune cell recruitment and positive correlation with cell cycle and DNA replication pathways. [file Image4.tif]

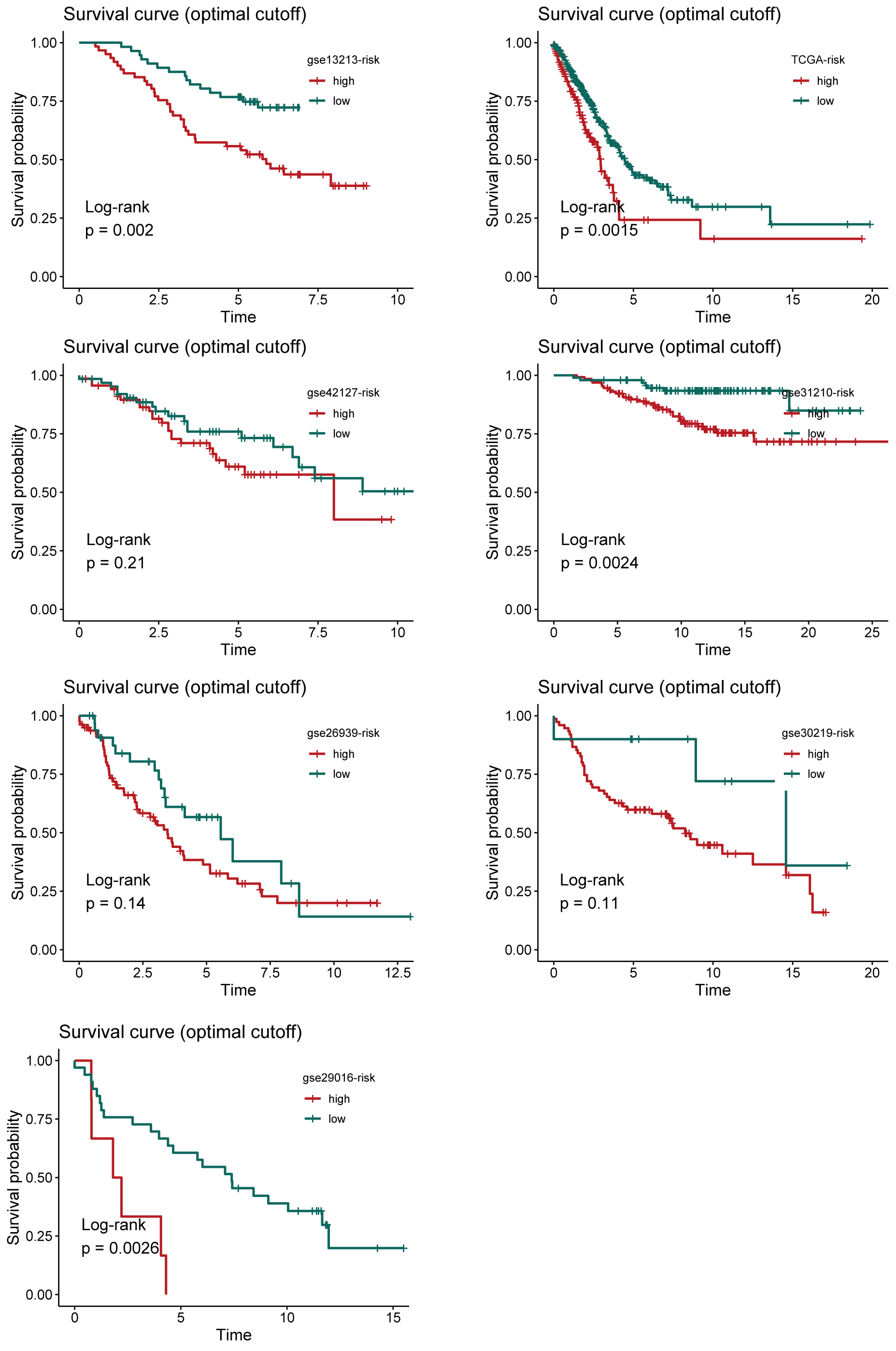

Supplement: Supplementary Figure 5 — Prognostic significance of DHCR7 across seven lung adenocarcinoma cohorts. [file Image5.tif]
